# Intrinsic in-plane nodal chain and generalized quaternion charge protected nodal link in photonics

**DOI:** 10.1038/s41377-021-00523-8

**Published:** 2021-04-15

**Authors:** Dongyang Wang, Biao Yang, Qinghua Guo, Ruo-Yang Zhang, Lingbo Xia, Xiaoqiang Su, Wen-Jie Chen, Jiaguang Han, Shuang Zhang, C. T. Chan

**Affiliations:** 1 Department of Physics, Hong Kong University of Science and Technology, Hong Kong, China; 2 College of Advanced Interdisciplinary Studies, National University of Defense Technology, Changsha, China; 3 Institute for Advanced Study, Hong Kong University of Science and Technology, Hong Kong, China; 4 Key Laboratory for Micro-Nano Optoelectronic Devices of Ministry of Education, School of Physics and Electronics, Hunan University, Changsha, China; 5 Institute of Solid State Physics and Department of Physics, Shanxi Datong University, Datong, China; 6 School of Physics & State Key Laboratory of Optoelectronic Materials and Technologies, Sun Yat-Sen University, Guangzhou, China; 7 Center for Terahertz Waves and College of Precision Instrument and Optoelectronics Engineering, Tianjin University and the Key Laboratory of Optoelectronics Information and Technology (Ministry of Education), Tianjin, China; 8 School of Physics & Astronomy, University of Birmingham, Birmingham, UK

**Keywords:** Optical physics, Metamaterials

## Abstract

Nodal lines are degeneracies formed by crossing bands in three-dimensional momentum space. Interestingly, these degenerate lines can chain together via touching points and manifest as nodal chains. These nodal chains are usually embedded in two orthogonal planes and protected by the corresponding mirror symmetries. Here, we propose and demonstrate an in-plane nodal chain in photonics, where all chained nodal lines coexist in a single mirror plane instead of two orthogonal ones. The chain point is stabilized by the intrinsic symmetry that is specific to electromagnetic waves at the Г point of zero frequency. By adding another mirror plane, we find a nodal ring that is constructed by two higher bands and links with the in-plane nodal chain. The nodal link in momentum space exhibits non-Abelian characteristics on a *C*_2_*T* - invariant plane, where admissible transitions of the nodal link structure are determined by generalized quaternion charges. Through near-field scanning measurements of bi-anisotropic metamaterials, we experimentally mapped out the in-plane nodal chain and nodal link in such systems.

## Introduction

Topological photonics has attracted a lot of attention recently^1,2^. The application of topological band theory to photonics not only opens the door to novel devices such as topological lasers^3–7^, but also stimulates the exploration of new topological phases, such as Floquet^8^ and high-order topological insulators^2,9^. Photonic systems offer a mature and highly flexible platform for topological phase discovery and realization^10–28^. We know that most electronic topological systems have their photonic counterparts, except for those depending on the intrinsic properties of fermion system, for example, 2D and 3D topological insulators^29,30^ with $T^2 = - 1$, where *T* is the time reversal operator. On the other hand, there are symmetries that are unique to electromagnetic (EM) waves, which can intrinsically protect the band degeneracies at isolated points in the momentum space^31–33^. Topological systems realized using such symmetries are uniquely “photonic”, having no counterparts in electronic or phononic systems.

Identifying nodal features in the band structure of topological materials, such as nodal points (Dirac or Weyl points) or nodal lines, can help to understand their topological characters. Among various topological features in momentum space, nodal chain^34–38^ is a special configuration of nodal lines^35,39–53^ where two nodal curves touch at isolated points. It is generally perceived that the two nodal lines should reside on two separate mirror planes, each protected by their corresponding mirror symmetries. The chain points are then found to be stabilized on their intersection lines. Here, making use of symmetries being intrinsic to electromagnetism, we theoretically propose and experimentally demonstrate a type of in-plane nodal chain, where the nodal lines are chained together in a single plane. In-plane chain points are usually fragile against perturbations, but the chain point in our system is protected by the electromagnetic intrinsic symmetry at the $\Gamma$ point unless a cut-off frequency is introduced due to artificial resonances. The in-plane nodal chain is uniquely stable in photonics due to the internal symmetries of the Maxwell equations and has no counterparts in other systems. This new type of nodal chain should widely exist in photonic systems, as long as additional symmetries such as mirror symmetries, protect the existence of nodal line branches. We also find a nodal ring linking with the in-plane nodal chain in our studied model with only two mirror symmetries, and the linked nodal structure exhibits non-Abelian features^54–59^ on a *C*_2_*T* - invariant plane^57^. The present study provides a realization of non-Abelian nodal links in the absence of Parity-Time (PT) symmetry^58^, and can be described using generalized quaternion charges of the photonic multi-band topology^54,55^. Finally, we experimentally demonstrate the in-plane nodal chain and nodal link with bi-anisotropic metamaterials.

## Results

We first use a generic two-band Hamiltonian to show the presence of the in-plane nodal chain. We then show that photonic intrinsic symmetry can stabilize the chain point. The Hamiltonian that exhibits the nodal line consists of two Pauli matrices as,

$$H = d_x\sigma _x + d_z\sigma _z$$

where $$d_x = k_yk_z$$, and

$$d_z = \left( {k_x^2 - k_y^4 - m} \right) + \left( {k_x^2 + k_z^2 - m} \right)$$

**Fig. 1:**
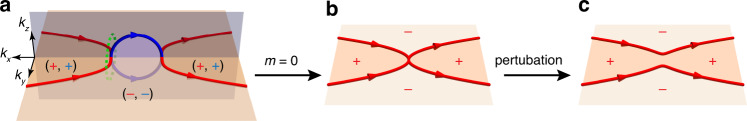
Transition from orthogonal nodal chain to in-plane nodal chain and separated nodal lines. **a** A normal nodal chain embedded in two orthogonal mirror planes. Green circle indicates the *π*_1_ homotopy loop. **b** The blue nodal ring can be shrunk continuously by tuning the system parameter *m* (see text), resulting in an in-plane nodal chain when *m* = 0. **c** Further perturbation finally gaps the chain point. For all panels, “±” indicates the signs of mirror symmetries eigenvalues, red color for *M*_*z*_ and blue color for *M*_*y*_. Within the framework of the two-band model, the eigenstates bounded inside the red (blue) degeneracy lines are of even (odd) parity, those outside are of odd (even) parity. Arrows indicate orientations of nodal lines with ${\mathbb{Z}}$ classified topological charges in a real two-band model

The nodal line structure, as a function of the parameter *m*, is shown in Fig. 1. For a value of *m* = +1, there are two chain points as shown in Fig. 1a. Going from left to right across the chain point, the two mirror-symmetry (both are represented by $\sigma _z$) eigenvalues change from $\left( {M_z,M_y} \right) = ( + 1, + 1)$ to $( - 1, - 1)$ on the intersection line of the two mirror planes (*k*_*y*_ = *k*_*z*_ = 0). For a two-band model with PT symmetry, the eigenfunctions are purely real, so that a loop encircling the chain point (or a general point on the nodal line) carries a ${\mathbb{Z}}$-valued charge, i.e., integer charges with positive or negative signs, which can be understood as the orientation defined on each nodal line^54^. Keeping the mirror symmetries intact, one can smoothly eliminate the vertical nodal ring (the blue one) by tuning *m* to zero, as shown in Fig. 1b, leading to an in-plane nodal chain. Meanwhile, the two out-of-plane chain points vanish concomitantly and merge into an in-plane chain point. The mirror eigenvalues of the eigenstates on the *k*_*y*_ = *k*_*z*_ = 0 line have the same (+) sign across the chain point, which indicate that the in-plane chain point is no longer stable since the symmetry eigenvalue sign change going across that point no longer exists. As shown in Fig. 1c, the in-plane chain point can be gapped by tuning *m* to negative values while preserving the mirror symmetry eigenvalues as labelled.

In photonic systems, however, the in-plane nodal chain can be stabilized by the intrinsic symmetry of electromagnetic wave at the Г point of zero frequency. In order to demonstrate the stable in-plane chain point, we start with an effective model of bi-anisotropic material, where inversion symmetry is explicitly broken, but two mirror symmetries preserve the nodal lines, one for the in-plane nodal chain and the other for the nodal ring that contributes to the nodal link. The effective constitutive parameters for the bi-anisotropic metamaterial under consideration (explicit design and experimental realization will be discussed in subsequent sections) are:

$$\overleftrightarrow \varepsilon = \left[ {\begin{array}{*{20}{c}} \varepsilon & 0 & 0 \\ 0 & \varepsilon & 0 \\ 0 & 0 & {\varepsilon _b} \end{array}} \right],\,\overleftrightarrow \mu = \left[ {\begin{array}{*{20}{c}} 1 & 0 & 0 \\ 0 & 1 & 0 \\ 0 & 0 & \mu \end{array}} \right],\,\overleftrightarrow \varsigma = \left[ {\begin{array}{*{20}{c}} 0 & 0 & 0 \\ 0 & 0 & 0 \\ { - i\chi } & { - i\chi } & 0 \end{array}} \right],\,\overleftrightarrow \xi = \left[ {\begin{array}{*{20}{c}} 0 & 0 & {i\chi } \\ 0 & 0 & {i\chi } \\ 0 & 0 & 0 \end{array}} \right].$$

The matrix elements depend on frequency and the structural parameters of the metamaterials, and take the form of $\varepsilon = \varepsilon _b + \frac{1}{{\omega _0^2 - \omega ^2}}\frac{{l^2}}{L}$, $\mu = 1 + \frac{2}{{\omega _0^2 - \omega ^2}}\frac{{\omega^2 {A}^2}}{L}$, $\chi = \frac{\omega }{{\omega _0^2 - \omega ^2}}\frac{{Al}}{L}$, where *ω*_*0*_ is a structure-induced resonance frequency, *ε*_*b*_ is the permittivity of substrate material, *L* is the effective inductance, *l* and *A* are the effective length and area of resonators. The bi-anisotropic metamaterial’s response to electromagnetic waves is given by Maxwell equations as $\nabla \times {\mathrm{E}} = - \partial _{\mathrm{t}}\left( {\overleftrightarrow \mu H + \overleftrightarrow \varsigma E} \right)$, $\nabla \times {\mathrm{H}} = \partial _{\mathrm{t}}(\overleftrightarrow \varepsilon {\mathrm{E}} + \overleftrightarrow \xi {\mathrm{H}})$.

**Fig. 2:**
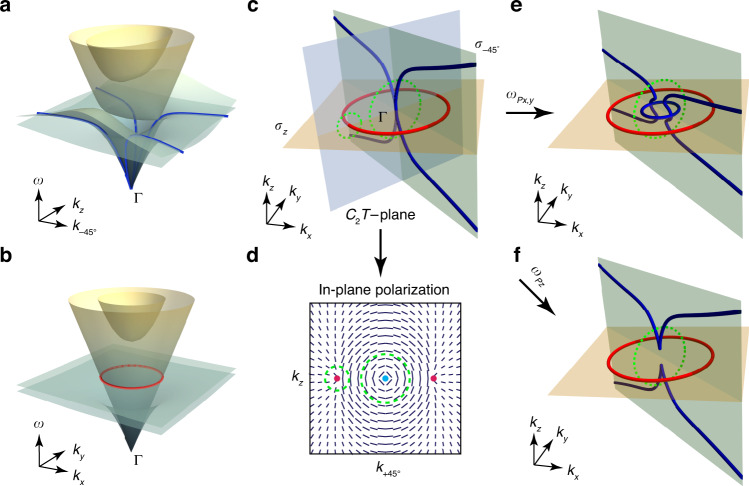
In-plane nodal chain and nodal link in bi-anisotropic metamaterials. **a** Band structure of the bi-anisotropic effective medium model in the space of [*k*_−45°_, *k*_*z*_, *ω*]. The blue lines are band degeneracies. Parameters adopted are *ω*_0_ = 2, *A* = 0.5, *l* = *L* = 1. **b** Band structure with *k*_*z*_ = 0, nodal ring is shown in red. **c** Nodal link in momentum space, in-plane nodal chain is shown in blue and the red circle is a nodal ring. Green circles indicate the *π*_1_ homotopy loops in the *C*_2_*T* - invariant plane. **d** The projected polarization states of the 2nd band at the *C*_2_*T* - invariant plane, the red dots are cut-positions of the nodal ring, blue dot is the in-plane nodal chain point at the Γ point. Green circles indicate *π*_1_ loops encircling different degeneracy nodes as in **c**. **e** The breaking of chain point with an in-plane plasmonic resonance, where a new nodal ring (blue) emerges on the *k*_*z*_ = 0 plane. The nodal ring connects the two nodal line branches as a consequence of the −1 non-Abelian charge accumulated along the green loop. **f** The breaking of chain point with z direction plasmon resonance. No new nodal structure shows up since the green loop still encircles two nodal lines and the −1 charge remains conserved

The energy bands of the proposed bi-anisotropic material can be studied with these effective constitutive parameter matrices. In Fig. 2a, band dispersions are calculated on the diagonal mirror plane of $k_x = - k_y$, where degeneracy lines are found between the 1st and 2nd bands and are highlighted as blue curves. As frequency decreases, the equi-frequency surfaces shrink, and the degenerate lines move closer to each other which finally merge at zero frequency at the Г point. By showing the degeneracy lines in the full momentum space, we can see from Fig. 2c that a pair of nodal lines (blue curves) touches each other at the Γ point. These nodal lines are embedded in a single plane of mirror symmetry, and as such, they are concrete examples of in-plane nodal chains we have referred to. It is important to note that the chain point is robust to perturbations due to the stable degeneracy of electromagnetic wave at zero frequency.

By examining the band structure on the other mirror plane (*k*_*z*_ = 0), a degeneracy ring (marked in red) between the 2nd and 3rd bands is found as shown in Fig. 2b. The nodal ring is induced by the electric resonance and appears at the frequency of *ω*|_*ε* = 0_, where a dispersionless longitudinal mode (flat band) appears and intersects the propagating transverse mode. More interestingly, the in-plane nodal chain (blue) threads through the nodal ring (red) in the momentum space as shown in Fig. 2c. A nodal link is thus constructed by three adjacent bands which give enough freedom to define non-Abelian charges. The non-Abelian charges represent the frame rotations of a set of real eigenfunctions, which form the elements of non-Abelian (generalized) quaternion groups. The charges characterize the band degeneracies and explain the protection of the global nodal structure that will be discussed later. The peculiar nodal link in Fig. 2c can be analytically described with the effective medium model (supplementary information 3), and linear dispersions of the nodal structures can be checked by considering *k*·*p* expansions (Supplementary information 4).

The nodal link in momentum space can be characterized by non-Abelian topological charges. We consider the first homotopy group (π_1_) loop encircling the chain point in the *C*_2_*T* - invariant plane, as indicated in Fig. 2c with a green circle. In the *C*_2_*T* - invariant plane, the photonic bands have real eigenvectors, and at each *k*-point, the set of eigenvectors defines a frame which rotates as the *k*-point goes along the green-colored loop. Such an eigenvector frame rotation can be described using generalized quaternion charges *n*_*Г*_ (*Г* indicating the π_1_ loop), which extend the notion of quaternion charges to system with more than three bands and account for the multi-band topology^54,55^. Degeneracy between the *j*th and (*j* + 1)th bands can be characterized as the non-Abelian charge of *n*_*Г*_ = *g*_*j*_ (see Supplementary information 5 for definition). In particular, frame rotation angles of 0 and 2π are distinguished by *n*_*Г*_ = +1 and −1, with −1 being the non-trivial case. The non-Abelian topological charge of −1 is uniquely interesting as it describes a topological character of multiple bands that cannot be described using the Berry phase quantization of individual bands. Since the non-Abelian topological charges are closely related to the rotation of the eigen polarization states, we show the projected polarization states (*E*_+45˚_, *E*_*z*_) of the 2nd band on the *C*_2_*T* - invariant plane in Fig. 2d, which illustrate the polarization rotation around the chain point (blue dot) as well as the nodal ring intersections in the *C*_2_*T* - invariant plane (red dots). As shown in Fig. 2d, a winding phase of 2π is found for the larger green loop encircling the chain point (blue dot). Such a winding pattern is akin to the order-parameter field in the vicinity of a 2π disclination line defect in biaxial nematic liquid crystals^60,61^, which can be characterized by a quaternion charge of −1 that describes 3D rotations. The 2π winding phase here indicates the generalized quaternion charge of *n*_*Г*_ = −1, a generalization to higher dimension as we are considering 5 bands together (see detail of non-Abelian charges calculation in supplementary information 5). The non-trivial topological charge of *n*_*Г*_ = −1 forbids the annihilation or breaking of the encircled nodal chain branches and plays an essential role in protecting the global nodal link structure in momentum space. The winding phase around the nodal ring intersection (red dots encircled by the small green circle in Fig. 2d) is found to be π, which corresponds to the generalized quaternion charge of *n*_*Г*_ = ±*g*_2_ that gives a characterization of a degeneracy node between the 2nd and 3rd bands.

The notion of non-Abelian charges can elegantly explain or predict admissible transitions of the nodal link as system parameter changes. The in-plane chain point at Γ is stable against perturbations that do not introduce a cut-off frequency which gaps the Γ point. In order to demonstrate the transition rule of the nodal link, we introduce plasmon resonances to the bi-anisotropic model, which gap the Γ point, pushing the nodal lines away from zero frequency. Two configurations of artificial plasmon resonances are considered for illustration purpose, one with resonance directions aligned with x and y axes, and the other along the z direction. In both cases, two mirror symmetries are maintained so as to protect the nodal lines. In Fig. 2e, we show the nodal structures in momentum space with in-plane artificial resonances along the x and y directions. The artificial resonances introduce non-zero energy (or mass) to the Hamiltonian at the Γ point. The chain point is now broken, however, in contrary to the two-band model in Fig. 1b, the nodal lines encircled by the green loop in Fig. 2e cannot just disappear, as a consequence of the conservation of −1 generalized quaternion charge. A new nodal ring (blue) has to emerge so as to conserve the charge of −1 and connects the two nodal line branches. In a different configuration, artificial resonance is introduced along the *k*_*z*_ direction in Fig. 2f. The chain point is also broken by the resonance, but in contrary to the previous case, the nodal chain here is allowed to break along *k*_*z*_ direction, and no new nodal structures are required to emerge. The transition mechanism can be understood from the schematic in Fig. 2f, where we can find that the two separated nodal line branches are still encircled by the green circle, and the −1 non-Abelian charge remains conserved. In addition, the protection by non-Abelian charges when plasmon resonance is introduced can again be explained by the rotation of eigen polarization states on the *C*_2_*T* - invariant plane (as in the case of Fig. 2d), and detail discussions are shown in supplementary information 6.

**Fig. 3:**
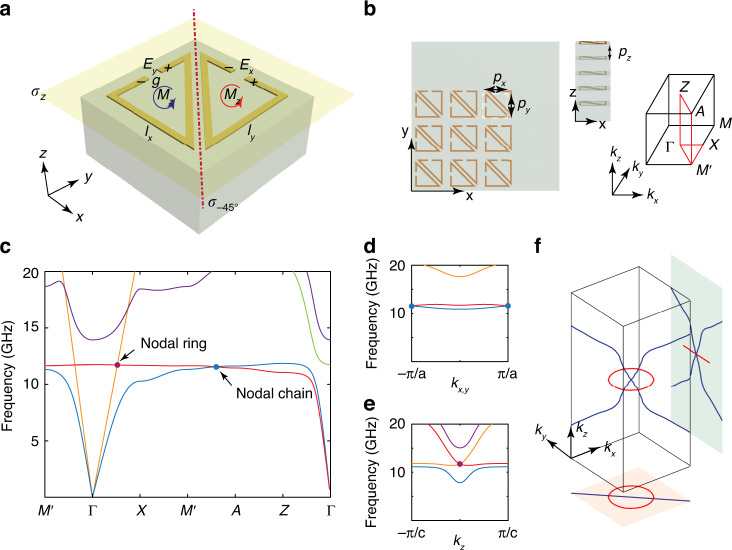
Realistic bi-anisotropic metamaterials with in-plane nodal chain and nodal link. **a** Schematic of the bi-anisotropic metamaterial. Split-ring resonators are arranged on the *σ*_*z*_ mirror plane. The resonators also possess another mirror symmetry of *σ*_*-45˚*_ as indicated by red dashed line. Side length of the resonator is *l*_*x*,*y*_ = 3.3 mm, and resonance gap is *g* = 0.5 mm. **b** The units are repeated to form 3D metamaterials, with lattice constants *p*_*x*_ = 4.5 mm, *p*_*y*_ = 4.5 mm and *p*_*z*_ = 2 mm. The Brillouin zone of the metamaterials is shown to the right. **c** Numerically calculated band structures, with degenerate points from in-plane nodal chain and nodal ring marked as blue and red dots, respectively. **d**, **e** Linear band dispersions along *k*_*x,y*_/*k*_*z*_ directions around the blue / red dot marked in **c**. **f** Nodal link in 3D momentum space corresponding to metamaterial shown in **b**, and projections of the nodal link onto the *k*_*y*_−*k*_*z*_ (green) and *k*_*x*_−*k*_*y*_ (orange) planes

Next, we demonstrate the proposed model experimentally in the microwave regime with a class of metallic metamaterials which exhibits topological line nodes^58,62,63^. A non-centrosymmetric meta-structure design is shown in Fig. 3a, where two planar split-ring triangular resonators are placed on the z = 0 mirror plane. Another mirror plane can be found along the diagonal direction as indicated by red dashed line. In the long wavelength limit, the meta-structure exhibits bi-anisotropic couplings as described by the effective medium parameters shown above. The driving electric fields induce currents running along *x* and *y* directions, and the “split-ring” configuration results in a magnetic moment along the *z* direction as schematically shown in Fig. 3a. In a reciprocal manner, the out of plane magnetic component *H*_*z*_ of incoming electromagnetic wave will induce in-plane polarizations of *P*_*x*_ and *P*_*y*_ accordingly (supplementary information 1–2). The resonating units are arrayed periodically as shown in Fig. 3b. They collectively contribute to the bi-anisotropic response of the metamaterials.

By utilizing the CST microwave studio numerical package, photonic band dispersions of the metamaterial structure in Fig. 3a are calculated and shown in Fig. 3c. A degeneracy point is found along M′ - A direction (blue dot) embedded in the diagonal mirror plane, which originates from the in-plane nodal chain. Additional degeneracy points of a nodal ring can be found along Γ - X/M′ directions (e.g., red dot) at *k*_*z*_ = 0 plane. The linear band dispersions at these marked degenerate points can be further examined along *k*_*x,y*_ and *k*_*z*_ directions, as shown in Fig. 3d, e (blue and red dots), respectively.

The nodal lines in momentum space formed by the intersections of the lowest three bands of the meta-structure are shown in Fig. 3f, where the in-plane nodal chain (blue) and nodal ring (red) are found on two perpendicular mirror planes. The in-plane nodal chain is protected by the intrinsic symmetry at Г point and extends to Brillouin zone boundaries. The nodal chain threads through the nodal ring and a nodal link constructed by three adjacent bands is thus formed. The projections of the nodal lines on the *k*_*x*_ − *k*_*y*_ plane (orange) and *k*_*y*_ − *k*_*z*_ plane (green) are also shown in Fig. 3f.

**Fig. 4:**
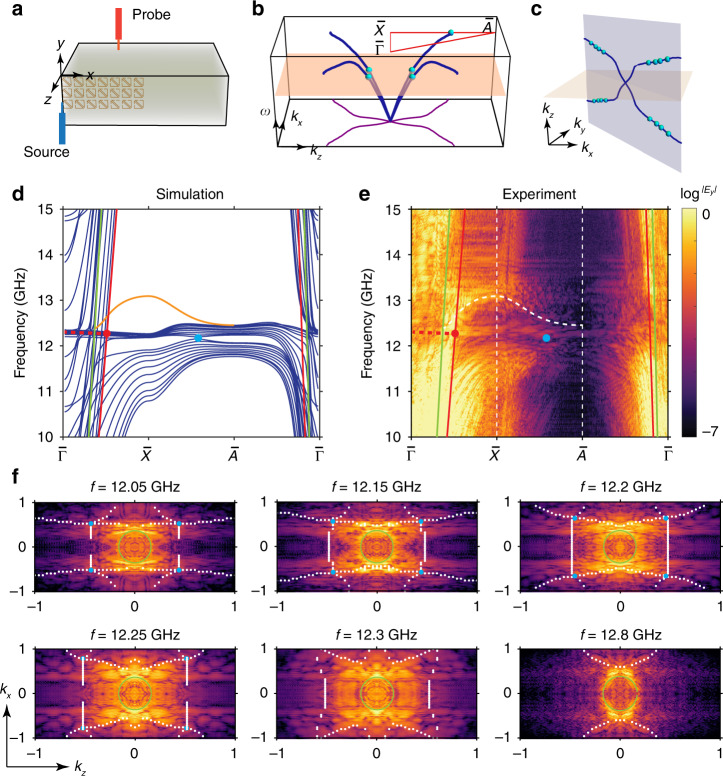
Experimental mapping of in-plane nodal chain within bi-anisotropic metamaterials. **a** Schematic of bi-anisotropic metamaterials for side surface (xz plane) scanning. Source and probe antennas are indicated in blue and red, respectively. **b** Calculated in-plane nodal chain (blue curves) dispersion, and its projection onto the *k*_*x*_ − *k*_*z*_ plane as the in-plane nodal chain (purple curves). Cyan dots show the degenerate points on one particular equi-frequency plane. Red triangle shows the path for the band spectra shown in **d**, **e**. **c** In-plane nodal chain in momentum space, blue lines are calculated results and cyan dots are from measurement results of **f**. **d**, **e** Calculated and experimentally mapped band spectra along $\bar \Gamma$ - $\overline {\mathrm{X}}$ - $\overline {\mathrm{A}}$ - $\bar \Gamma$. For both simulation and experimental results, the blue dot marks the point where the nodal chain meets the Brillouin zone boundary on the $\overline {\mathrm{X}}$ - $\overline {\mathrm{A}}$ line. Surface mode is indicated with orange curve in **d** and white dashed curve in **e**. Nodal ring projection is indicated with red dashed line. Light cone for air and effective substrate (*ε*_*b*_ ≈ 1.95) are shown with green and red solid lines, respectively. **f** Experimentally mapped EFCs for different frequencies. Calculated EFCs are marked with white dots. Blue dots indicate the nodal points at the corresponding frequencies. Green rings are the EFCs of light cone for air

We then fabricate the sample (80 stacked PCB layers along the z direction; 100 × 10 unit cells in the xy plane) and experimentally characterize these nodal lines. An experimental configuration is shown in Fig. 4a, where the side surface (xz plane) is configured for raster-scanning with a near-field probe antenna (red antenna). A point source (blue antenna) is placed at the corner of the sample and the field distribution on the side surface is to be measured. The measured field distributions can be subsequently Fourier transformed to obtain the projected band information in momentum space. The calculated in-plane nodal chain corresponding to the experimental configuration is shown in the space of [*k*_*x*_, *k*_*z*_, *ω*] in Fig. 4b, which projects onto the *k*_*x*_–*k*_*z*_ plane as the purple curve. The equi-frequency contours (EFCs) intersect with the blue curves as four cyan points of bands degeneracies, as will be experimentally measured later. The in-plane nodal chain is also shown in 3D momentum space of Fig. 4c as blue lines.

In Fig. 4d, e, we show respectively the calculated and experimentally measured band spectra along the lines $\bar \Gamma$ - ${\bar{\mathrm X}}$ - ${\bar{\mathrm A}}$ - $\bar \Gamma$ defined in Fig. 4b. A touching point of projected bands, marked with a blue dot, is observed along ${\bar{\mathrm X}}$ - ${\bar{\mathrm A}}$ as shown in Fig. 4e. The measured position agrees with the calculated band touching point marked as the blue dot in Fig. 4d. This particular touching point of band projection is the cut position of in-plane nodal chain at the Brillouin zone boundary (${\bar{\mathrm X}}$ - ${\bar{\mathrm A}}$) as illustrated in Fig. 4b. Surface modes can also be found for the side surface configuration, which are indicated with the orange curve in Fig. 4d and white dotted curve in Fig. 4e. Meanwhile, indications of the nodal ring degeneracies can also be found in the projected band spectra on side surface. The nodal ring is located at the *k*_*z*_ = 0 plane and projected onto the $\bar \Gamma$ - ${\bar{\mathrm X}}$ line of Fig. 4b for the side surface configuration. In the $\bar \Gamma$ - ${\bar{\mathrm X}}$ part of Fig. 4d, e, longitudinal flat band projections are indicated with red dashed lines, and the transverse mode for *k*_*z*_ = *k*_*y*_ = 0 (also the light cone for substrate) is plot as red solid line. The degeneracy point between them is indicated as red dot, which is the nodal ring degeneracy projection along $\bar \Gamma$ - ${\bar{\mathrm X}}$. We further show the mapped EFCs in Fig. 4f with respect to several frequencies from *f* = 12.05 GHz to *f* = 12.3 GHz. At each frequency, the boundaries of projected bands from the calculations are shown as white dots. Blue dots are the EFCs-cut nodal points explained in Fig. 4b and shown together in Fig. 4c as cyan dots. At a higher frequency of *f* = 12.8 GHz, the EFCs of surface mode is measured and indicated with white dashed lines in the last panel of Fig. 4f (more discussions of surface states in supplementary information 7).

**Fig. 5:**
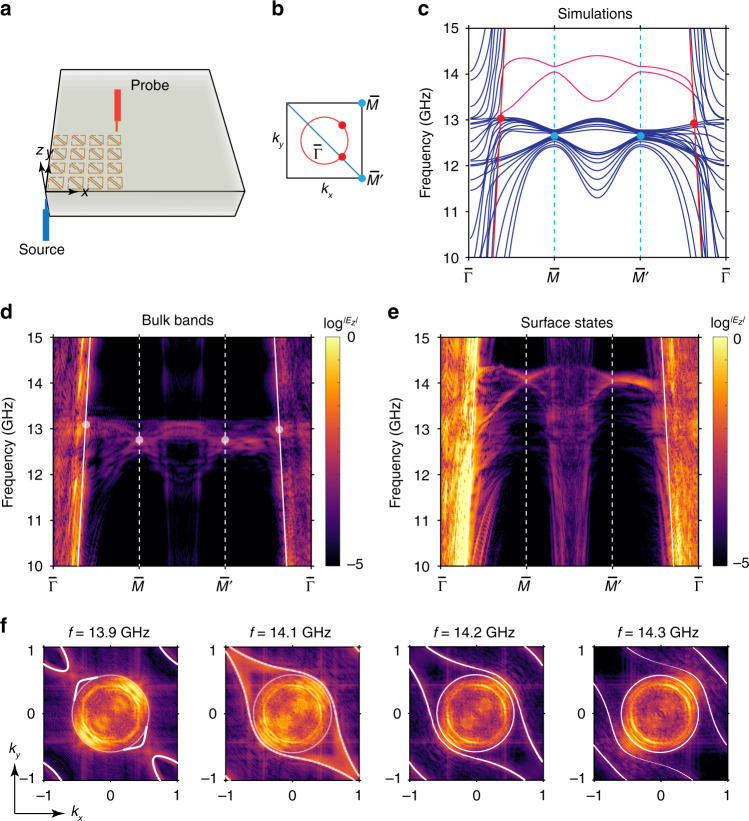
Experimental measurements of nodal lines and surface modes at the top surface. **a** Schematic of the experimental measurement configuration on the top surface (xy plane). The source and probe antennas are colored in blue and red, respectively. **b** Surface Brillouin zone of the top surface. Blue line indicates the projection of in-plane nodal chain and red circle is the nodal ring projection. **c** Calculated projected band spectra for the top surface. Surface modes are indicated with red color. Blue (Red) dots indicate the degenerate points on the in-plane nodal chain (nodal ring), as also illustrated in **b.** Substrate light cone is shown as red solid lines (*ε*_*b*_ ≈ 1.8). It should be noted that the slight difference of effective permittivity from above measurement is due to the sample compactness in two configurations. **d**, **e** Measured bulk bands and surface states on the top surface, respectively. Degeneracy positions of nodal chain (ring) are indicated with white dots in **d**. Substrate light cones are shown as white solid lines. **f** Measured and calculated (white lines) EFCs of surface states at the frequencies of *f* = [13.9, 14.1, 14.2, 14.3] GHz. EFCs for substrate light cone are shown as white circles

In a different experimental configuration, we measured the top surface (xy plane) field distributions of the layered metamaterials in Fig. 5a. We used a sample containing 100 × 100 unit cells in the xy plane and 10 layers in the z direction. The surface Brillouin zone is shown in Fig. 5b, with the blue line indicating the projected in-plane nodal chain and the red circle marking the nodal ring. The projected band dispersion along the z direction is shown in Fig. 5c, where the projected bulk bands have degeneracies at ${\bar{\mathrm M}}$ and ${\bar{\mathrm M}}^\prime$ points around *f* = 12.5 GHz (indicated with blue dots). These degeneracies at ${\bar{\mathrm M}}/{\bar{\mathrm M}}^\prime$ originate from the in-plane nodal chain as explained and marked in Fig. 5b. We note that the projected bulk bands along $\bar \Gamma$ - ${\bar{\mathrm M}}^\prime$ possess no energy gap around 12.5 GHz in contrast to the bands along $\bar \Gamma$ - ${\bar{\mathrm M}}$, which is a result of the in-plane nodal chain projection long −45˚ diagonal direction (blue line in Fig. 5b). Additional degeneracy positions of projected bands can be seen along $\bar \Gamma$ - $\overline {\mathrm{M}} /\overline {\mathrm{M}} ^\prime$ as red dots. These crossings are between the longitude modes (nearly flat bands) and transverse modes (close to light cone in red), which marks the nodal ring degeneracies. Surface modes are found above the bulk bands, as indicated with red lines.

By tuning the source antenna position along the z direction, the bulk and surface states can be selectively excited as demanded. During the measurement of bulk bands, a thin layer of dielectric is used to cover the top of the layered metamaterials, and the modified boundary condition eliminates the high frequency surface modes. We show the measured bulk bands in Fig. 5d. The degeneracies at ${\bar{\mathrm M}}$ and ${\bar{\mathrm M}}^\prime$ points are experimentally observed for the in-plane nodal chain and indicated with white dots. The crossings between transverse and longitude modes are also observed and marked with white dots near the light cone (white straight lines), which can be identified as the nodal ring projection in Fig. 5b. Removing the dielectric layer covering the top, the surface modes can be observed. The measured surface state bands are shown in Fig. 5e, and surface modes are found at higher frequencies as in good accordance with the calculated results of Fig. 5c. We also show the EFCs of surface modes with respect to different frequencies in Fig. 5f, where calculated (white lines) and measured results can also be found with good accordance.

## Discussion

The photonic in-plane nodal chain realized in our system is protected by mirror symmetry and electromagnetic intrinsic degeneracy at zero frequency. The latter is robust as long as no plasmon resonance is introduced to gap the Γ point, such as introducing metallic wires in metamaterials. The nodal link constructed between three consecutive bands can be characterized with non-Abelian topological charges, which provide extra protections to the nodal structure at the *C*_2_*T* - invariant plane as illustrated in Fig. 2e, f.

The in-plane nodal chain (even the nodal link) should widely exist in photonic systems, and the bi-anisotropic metamaterials studied here serve as a clean and simple model with the necessary and sufficient symmetry conditions to manifest the in-plane nodal chain and nodal link (supplementary information 8, 9). Breaking the mirror symmetry would transform the nodal lines into Weyl points (supplementary information 10). Our metamaterial system thus provides a platform for studying topological phase transitions. The novel structure of linked in-plane nodal chain and nodal ring in our demonstrated system could also find applications in designing novel functioning photonic devices.

## Materials and methods

Simulations are carried out with CST microwave studio and Comsol Multiphysics. The sample is fabricated with Printed Circuit Board techniques. For top surface measurement, a 10-layer sample with 100 × 100 unit cells was fabricated, the whole sample size is 45 cm by 45 cm by 4 cm. For the side surface measurement, 80 × 100 unit cells are configurated, and the thickness of sample is 4.5 cm (10 unit cells). The experimental measurements are conducted with microwave near field scanning system, where a vector network analyzer (VNA) is used to connect both the source and probe antennas. The field information is Fourier transformed to achieve the projected bands and EFCs.

## Supplementary information

Supplementary information for Intrinsic in-plane nodal chain and generalized quaternion charge protected nodal link in photonics
