# Supplementary material for: Intrinsic in-plane nodal chain and generalized quaternion charge protected nodal link in photonics
Source: Light Sci Appl. 2021 Apr 15;10:83. doi: 10.1038/s41377-021-00523-8 (PMC8050084; doi:10.1038/s41377-021-00523-8)
Supplement: Supplementary file 1 — Supplementary information for Intrinsic in-plane nodal chain and generalized quaternion charge protected nodal link in photonics [file 41377_2021_523_MOESM1_ESM.docx]

**Supplementary Information for**

**Intrinsic in-plane nodal chain and generalized quaternion charge protected nodal link in photonics**

Dongyang Wang^1†^, Biao Yang^1,2†^, Qinghua Guo^1,3†^, Ruo-Yang Zhang^1^, Lingbo Xia^4^, Xiaoqiang Su^5^, Wen-Jie Chen^6^, Jiaguang Han^7*^, Shuang Zhang^8*^, C. T. Chan^1*^

1. Department of Physics, Hong Kong University of Science and Technology, Hong Kong, China.
2. College of Advanced Interdisciplinary Studies, National University of Defense Technology, Changsha 410073, China.
3. Institute for Advanced Study, Hong Kong University of Science and Technology, Hong Kong, China.
4. Key Laboratory for Micro-Nano Optoelectronic Devices of Ministry of Education, School of Physics and Electronics, Hunan University, Changsha 410082, China.
5. Institute of Solid State Physics and Department of Physics, Shanxi Datong University, Datong 037009, China.
6. School of Physics & State Key Laboratory of Optoelectronic Materials and Technologies, Sun Yat-Sen University, Guangzhou 510275, China.
7. Center for Terahertz Waves and College of Precision Instrument and Optoelectronics Engineering, Tianjin University and the Key Laboratory of Optoelectronics Information and Technology (Ministry of Education), Tianjin 300072, China.
8. School of Physics & Astronomy, University of Birmingham, Birmingham, B15 2TT, UK.

*Correspondence to: jiaghan@tju.edu.cn; s.zhang@bham.ac.uk; phchan@ust.hk

†These authors contributed equally to this work.

**1. Description of metamaterials response**

The split ring resonators (SRRs) inside the unit cell of the metamaterials can be described as components of *LC* circuits, and they give a resonance frequency of $\omega_{0}=\frac{1}{\sqrt{LC}}$. The electromagnetic (EM) response is described according to,

$\frac{\partial I}{\partial t}L=-\frac{\partial B}{\partial t}A$,

$\frac{q}{C}=El$,

where *L* and *C* are the effective inductance and capacitance, *l* and *A* are the effective length and area of resonators, respectively. The bi-anisotropic couplings are then introduced through polarization and magnetization as:

$$M=AI=\frac{A}{l}\frac{dP}{dt}$$

The effective EM response of metamaterials can thus be formulated into the following matrix formalism:

$$\left[ \begin{matrix} -\omega\varepsilon_{b}\varepsilon_{0} & 0 & 0 & 0 & k_{z} & -k_{y} & 0 & 0 & -i\frac{l}{A} & 0 \\ 0 & -\omega\varepsilon_{b}\varepsilon_{0} & 0 & -k_{z} & 0 & k_{x} & 0 & 0 & 0 & -i\frac{l}{A} \\ 0 & 0 & -\omega\varepsilon_{b}\varepsilon_{0} & k_{y} & -k_{x} & 0 & 0 & 0 & 0 & 0 \\ 0 & -k_{z} & k_{y} & -\omega\mu_{0} & 0 & 0 & 0 & 0 & 0 & 0 \\ k_{z} & 0 & -k_{x} & 0 & -\omega\mu_{0} & 0 & 0 & 0 & 0 & 0 \\ -k_{y} & k_{x} & 0 & 0 & 0 & -\omega\mu_{0} & 0 & 0 & -\omega& -\omega\\ 0 & 0 & 0 & 0 & 0 & 0 & -\omega\frac{L\omega_{0}^{2}}{l^{2}} & 0 & i\frac{L\omega_{0}^{2}}{lA} & 0 \\ 0 & 0 & 0 & 0 & 0 & 0 & 0 & -\omega\frac{L\omega_{0}^{2}}{l^{2}} & 0 & i\frac{L\omega_{0}^{2}}{lA} \\ i\frac{l}{A} & 0 & 0 & 0 & 0 & -\omega& -i\frac{L\omega_{0}^{2}}{lA} & 0 & -\frac{L\omega}{A^{2}} & 0 \\ 0 & i\frac{l}{A} & 0 & 0 & 0 & -\omega& 0 & -i\frac{L\omega_{0}^{2}}{lA} & 0 & -\frac{L\omega}{A^{2}} \end{matrix} \right]\left[ \begin{matrix} E_{x} \\ E_{y} \\ E_{z} \\ H_{x} \\ H_{y} \\ H_{z} \\ P_{x} \\ P_{y} \\ M_{z-x} \\ M_{z-y} \end{matrix} \right]=0$$

The response equations are further simplified and transformed into a generalized eigenvalue problem

$$M\varphi=\omega N\varphi$$

with the basis as,

$$\varphi=\left[ \begin{matrix} E_{x} & E_{y} & E_{z} & H_{x} & H_{y} & H_{z} & P_{x} & P_{y} & M_{z-x} & M_{z-y} \end{matrix} \right]^{T}$$

and the coefficient matrices *M* and *N* as,

$$M=\left[ \begin{matrix} 0 & 0 & 0 & 0 & k_{z} & -k_{y} & 0 & 0 & -i\frac{l}{A} & 0 \\ 0 & 0 & 0 & -k_{z} & 0 & k_{x} & 0 & 0 & 0 & -i\frac{l}{A} \\ 0 & 0 & 0 & k_{y} & -k_{x} & 0 & 0 & 0 & 0 & 0 \\ 0 & -k_{z} & k_{y} & 0 & 0 & 0 & 0 & 0 & 0 & 0 \\ k_{z} & 0 & -k_{x} & 0 & 0 & 0 & 0 & 0 & 0 & 0 \\ -k_{y} & k_{x} & 0 & 0 & 0 & 0 & 0 & 0 & 0 & 0 \\ 0 & 0 & 0 & 0 & 0 & 0 & 0 & 0 & i\frac{L\omega_{0}^{2}}{lA} & 0 \\ 0 & 0 & 0 & 0 & 0 & 0 & 0 & 0 & 0 & i\frac{L\omega_{0}^{2}}{lA} \\ i\frac{l}{A} & 0 & 0 & 0 & 0 & 0 & -i\frac{L\omega_{0}^{2}}{lA} & 0 & 0 & 0 \\ 0 & i\frac{l}{A} & 0 & 0 & 0 & 0 & 0 & -i\frac{L\omega_{0}^{2}}{lA} & 0 & 0 \end{matrix} \right]$$

$$N=\left[ \begin{matrix} \varepsilon_{b}\varepsilon_{0} & 0 & 0 & 0 & 0 & 0 & 0 & 0 & 0 & 0 \\ 0 & \varepsilon_{b}\varepsilon_{0} & 0 & 0 & 0 & 0 & 0 & 0 & 0 & 0 \\ 0 & 0 & \varepsilon_{b}\varepsilon_{0} & 0 & 0 & 0 & 0 & 0 & 0 & 0 \\ 0 & 0 & 0 & \mu_{0} & 0 & 0 & 0 & 0 & 0 & 0 \\ 0 & 0 & 0 & 0 & \mu_{0} & 0 & 0 & 0 & 0 & 0 \\ 0 & 0 & 0 & 0 & 0 & \mu_{0} & 0 & 0 & 1 & 1 \\ 0 & 0 & 0 & 0 & 0 & 0 & \frac{L\omega_{0}^{2}}{l^{2}} & 0 & 0 & 0 \\ 0 & 0 & 0 & 0 & 0 & 0 & 0 & \frac{L\omega_{0}^{2}}{l^{2}} & 0 & 0 \\ 0 & 0 & 0 & 0 & 0 & 1 & 0 & 0 & \frac{L}{A^{2}} & 0 \\ 0 & 0 & 0 & 0 & 0 & 1 & 0 & 0 & 0 & \frac{L}{A^{2}} \end{matrix} \right]$$

Then the effective Hamiltonian can be written in Hermitian formalism as,

$$H=N^{-\frac{1}{2}}MN^{-\frac{1}{2}}$$

which satisfies,

$$H\varphi^{'}=E\varphi^{'}$$

with,

$$\varphi^{'}=N^{\frac{1}{2}}\varphi$$

**2. Effective constitutive parameters for the metamaterials**

The EM response of our metamaterials can be described by polarization and magnetization which are derived to be:

$$P_{x}=\frac{1}{\omega_{0}^{2}-\omega^{2}}\frac{l}{L}\left( E_{x}l+Ai\omega H_{z} \right)$$

$$P_{y}=\frac{1}{\omega_{0}^{2}-\omega^{2}}\frac{l}{L}\left( E_{y}l+Ai\omega H_{z} \right)$$

$$M_{z-x}=-i\frac{1}{\omega_{0}^{2}-\omega^{2}}\frac{\omega A}{L}\left( E_{x}l+Ai\omega H_{z} \right)$$

$$M_{y-x}=-i\frac{1}{\omega_{0}^{2}-\omega^{2}}\frac{\omega A}{L}\left( E_{y}l+Ai\omega H_{z} \right)$$

We then formulate the effective constitutive parameters as,

$\varepsilon=\left[ \begin{matrix} \varepsilon& 0 & 0 \\ 0 & \varepsilon& 0 \\ 0 & 0 & \varepsilon_{b} \end{matrix} \right]$, $\mu=\left[ \begin{matrix} 1 & 0 & 0 \\ 0 & 1 & 0 \\ 0 & 0 & \mu\end{matrix} \right]$, $\varsigma=\left[ \begin{matrix} 0 & 0 & 0 \\ 0 & 0 & 0 \\ -i\chi& -i\chi& 0 \end{matrix} \right]$, $\xi=\left[ \begin{matrix} 0 & 0 & i\chi\\ 0 & 0 & i\chi\\ 0 & 0 & 0 \end{matrix} \right]$

with,

$\varepsilon=\varepsilon_{b}+\frac{1}{\omega_{0}^{2}-\omega^{2}}\frac{l^{2}}{L}$, $\mu=1+\frac{2}{\omega_{0}^{2}-\omega^{2}}\frac{\omega^{2}A^{2}}{L}$, $\chi=\frac{\omega}{\omega_{0}^{2}-\omega^{2}}\frac{Al}{L}$

and Maxwell equations for the homogenized medium can be rewritten as,

$$\nabla\times E=-\frac{\partial}{\partial t}\left( \mu H+\varsigma E \right)$$

$$\nabla\times H=\frac{\partial}{\partial t}\left( \varepsilon E+\xi H \right)$$

The dispersions of effective medium parameters are shown in Fig. S1 below.


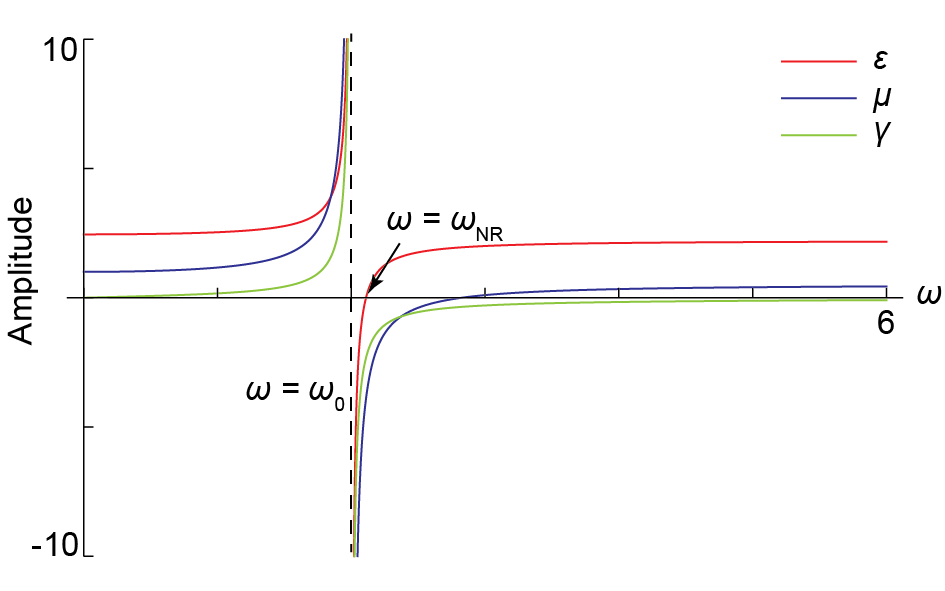


**Fig. S1 Dispersion of effective medium parameters.** The frequency dispersion of *ε*, *μ* and *γ* are plotted with red, blue and green colors, respectively. The *LC* resonance frequency *ω*_0_ is indicated with black dashed line, and the nodal ring frequency is marked as *ω*_NR_, where *ε* = 0 give a longitudinal resonance. Parameters uses in calculations are *ω*_0_ = 2, *A* = 0.5, *L* = 1, *l* = 1, *ε_b_* = 2.2. The same set of parameters are used through the following sections unless otherwise specified.

**3. Analytical description of nodal curves**

Based on the effective medium model, the band dispersions can be calculated. Two type of nodal curves are found as shown in Fig. S2. The nodal structure contains a nodal ring at $k_{z}=0$ plane, and an in-plane nodal chain located at the $k_{x}=-k_{y}$ plane that extends to infinity. They form a nodal link in 3D momentum space. The nodal curves can be described analytically as follows.

For the nodal ring, we substitute $k_{z}=0$ into the Maxwell equations and solve for the crossing bands (indexed as 1 and 2),

$\omega_{Ring}^{1}=\frac{\sqrt{k_{x}^{2}+k_{y}^{2}}}{\sqrt{\varepsilon_{b}}}$,$\omega_{Ring}^{2}=\frac{\sqrt{l^{2}+\varepsilon_{b}L\omega_{0}^{2}}}{\sqrt{\varepsilon_{b}L}}$

which gives the solution of nodal ring as:

$$k_{x}^{2}+k_{y}^{2}={l^{2}}/L+\varepsilon_{b}\omega_{0}^{2}$$

For the in-plane nodal chain, we take $k_{y}=-k_{x}$ and the effective medium model gives solutions of,

$\omega_{Nc}^{1}=\frac{\sqrt{2\varepsilon k_{x}^{2}+\varepsilon_{b}k_{z}^{2}}}{\sqrt{\varepsilon\varepsilon_{b}}}$, $\omega_{Nc}^{2}=\frac{\sqrt{2k_{x}^{2}+\mu k_{z}^{2}}}{\sqrt{\varepsilon\mu-2r^{2}}}$

Further considering the dispersion of effective material parameters, the exact band dispersion of two crossing bands are,

$\omega_{1}=\sqrt{\frac{2A^{2}k_{z}^{2}-l^{2}-L\left( 2k_{x}^{2}+k_{z}^{2}+\varepsilon_{b}\omega_{0}^{2} \right)+\sqrt{4\varepsilon_{b}L\omega_{0}^{2}\left( 2k_{x}^{2}+k_{z}^{2} \right)\left( 2A^{2}-L \right)+\left( 2A^{2}k_{z}^{2}-l^{2}-L\left( 2k_{x}^{2}+k_{z}^{2}+\varepsilon_{b}\omega_{0}^{2} \right) \right)^{2}}}{4A^{2}\varepsilon_{b}-2\varepsilon_{b}L}}$,

$\omega_{2}=\sqrt{\frac{l^{2}+L\left( 2k_{x}^{2}+k_{z}^{2}+\varepsilon_{b}\omega_{0}^{2} \right)-\sqrt{l^{4}+L^{2}\left( 2k_{x}^{2}+k_{z}^{2}-\varepsilon_{b}\omega_{0}^{2} \right)^{2}+2l^{2}L\left( -2k_{x}^{2}+k_{z}^{2}+\varepsilon_{b}\omega_{0}^{2} \right)}}{2\varepsilon_{b}L}}$,

The crossing positions satisfy $\omega_{1}=\omega_{2}$, which leads to solutions of,

$$k_{z}^{2}=\frac{1}{2A^{2}\varepsilon_{b}L\left( l^{2}+2k_{x}^{2}L \right)\omega_{0}^{2}}\left( -k_{x}^{2}\left( l^{2}+2k_{x}^{2}L-\varepsilon_{b}L\omega_{0}^{2} \right)\left( -l^{2}L+2A^{2}\left( l^{2}+\varepsilon_{b}L\omega_{0}^{2} \right) \right)\pm k_{x}\left( l^{2}+2k_{x}^{2}L+\varepsilon_{b}L\omega_{0}^{2} \right)\sqrt{k_{x}^{2}l^{4}L^{2}+4A^{4}k_{x}^{2}\left( l^{2}+\varepsilon_{b}L\omega_{0}^{2} \right)^{2}+4A^{2}l^{2}L\left( \varepsilon_{b}l^{2}\omega_{0}^{2}+k_{x}^{2}\left( \varepsilon_{b}L\omega_{0}^{2}-l^{2} \right) \right)} \right)$$

These branches of nodal lines are chained together at the *Γ* point (with zero frequency) as shown in Fig. S2a, the touching point dispersion is $k_{z}\approx2.57k_{x}^{1/2}+1.34k_{x}^{3/2}-0.26k_{x}^{5/2}+\ldots$, which indicates the non-linear touching of the chain point.


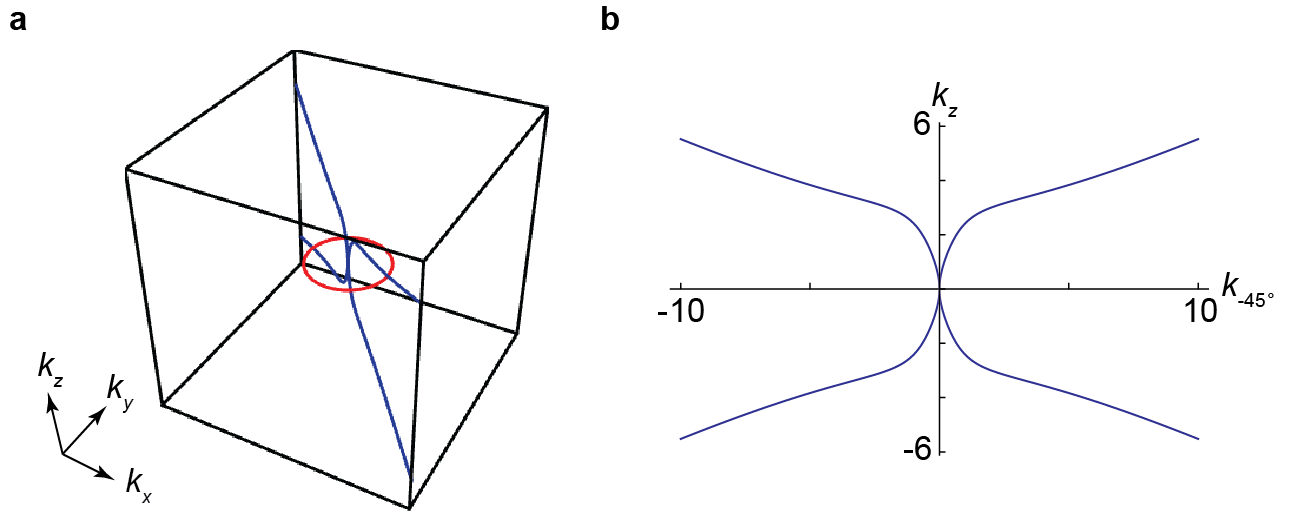


**Fig. S2 Nodal links and non-linear touching of in-plane nodal chain. a**. The nodal ring and in-plane nodal chain form a nodal link in 3D momentum space. **b**. The touching of two in-plane nodal chain branches.

**4. The *k* · *p* expansions around line nodes**

The expansions around nodal curve degeneracies possess linear dispersions along two directions. We verify this for some points on the nodal ring and in-plane nodal chain.

For the nodal ring, a point is selected at $\left[ k_{x}, k_{y}, k_{z} \right]=\left[ \sqrt{{l^{2}}/L+\varepsilon_{b}\omega_{0}^{2}}, 0, 0 \right]$, the effective Hamiltonian is,

$\left[ \begin{matrix} H_{11} & H_{12} \\ H_{21} & H_{22} \end{matrix} \right]$,

where,

$$H_{ij}=\sum_{m=x,y,z} V_{i}^{\dagger}\frac{dH}{dk_{m}}V_{j}$$

The calculated effective Hamiltonian is $H_{eff}=\left( a\sigma_{1}+b\sigma_{2} \right)dk_{z}+\frac{c}{2}\left( \sigma_{0}-\sigma_{3} \right)dk_{x}$ with *a* = -0.044, *b* = 0.07, *c* = 0.674 and *σ*_1,2,3_ are Pauli matrices. The linear dispersions are thus verified to be along *k_x_* and *k_z_* directions.

For the in-plane nodal chain embedded in *σ*-_45°_ plane, let us consider a point with coordinates of $\left[ k_{x}, k_{y}, k_{z} \right]=\left[ 1, -1, 2.735 \right]$. The local derivative of nodal chain at the selected point is denoted as $\eta={dk_{z}}/{dk_{-45^{\circ}}}\approx0.587$. The expansions are then calculated along three directions of *v*_1_ = d*k*-_45°_ + *ηdk*_z_, *v*_2_ = -*ηdk*-_45°_ + d*k_z_*, and *v*_3_ = d*k*_45°_, respectively. The effective Hamiltonian can be written as $H_{eff}=0.334\sigma_{0}dk_{v1}+\left( -0.008\sigma_{1}-0.094\sigma_{2}+0.002\sigma_{3} \right)dk_{45^{\circ}}+\left( 0.259\sigma_{0}+0.122\sigma_{1}-0.012\sigma_{2}-0.038\sigma_{3} \right)dk_{v2}$. The linear dispersions are along *k*_45°_ and *k_v_*_2_ directions.

**5. Generalized quaternion charges in bi-anisotropic metamaterials**

Although our studied system is non-centrosymmetric, there is a *C*_2_*T*-invariant plane that allows for the consideration of *N*-dimensional frame rotation of the eigenvectors *φ_n_*. The band topology can then be characterized by the defined frame. The space of *N*-dimensional Hamiltonian is expressed as^55^,

$$M_{N}=SO\left( N \right)/P\left( N \right)=Spin\left( N \right)/\bar{P}_{N}$$

where the first homotopy group of π_1_(*M_N_*) coincides with discrete group $\bar{P}_{N}$. The group of $\bar{P}_{N}$ is generated by (N-1) π rotations of [*e*_1_, *e*_2_, …,*e*_N-1_], where *e*_j-1_ corresponds to a π rotation in the [*x*_1_, *x_j_*] plane.

The rotation of frame along a closed path Г can be characterized by integrating the path-ordered Berry-Wilczek-Zee (BWZ) connection over all bands as,

$$R\left( \Gamma\right)=\bar{exp}\left( \oint A_{\Gamma}\left( k \right)\cdot dk \right)$$

with $\left[ A_{\Gamma}\left( k \right) \right]_{aj}^{i}=\left\langle u_{k}^{i} | {\partial_{k}}_{a} | u_{k}^{j} \right\rangle$, and *i*, *j*∈[1,2,…N].

The generalized quaternion charge can be calculated by lifting the connection basis of *SO*(*N*) to *Spin*(*N*), which provides the capability of distinguishing 2π rotation from 0. The generalized quaternion charge is thus formulated as,

$$n_{\Gamma}=\bar{R}\left( \Gamma\right)=\bar{exp}\left( \oint\bar{A_{\Gamma}}\left( k \right)\cdot dk \right)$$

where the lifted BWZ connection matrix is denoted as $\bar{A_{\Gamma}}\left( k \right)$.

For a *N*-band Hamiltonian, the *SO*(*N*) basis can be constructed according to,

$$\left( L_{ij} \right)_{ab}=-\delta_{ia}\delta_{jb}+\delta_{ib}\delta_{ja}$$

and the *Spin*(*N*) basis are,

$$t_{ij}=-\frac{1}{4}\left[ \Gamma_{i},\Gamma_{j} \right]$$

where *Г_i_* are anti-commutating Gamma matrices in *N* dimensions.

The π rotation can be formulated as,

$$e_{j-1}=e^{\pi t_{1j}}=2t_{1j}$$

and more general descriptions of the rotations are taken as,

$$g_{j}=\left\{ \begin{aligned} e_{1}, j=1 \\ e_{j-1}e_{j}, j\geq2 \end{aligned} \right.$$

The group $\bar{P}_{N}$ is formed of components as $\left[ \pm1,\pm g_{1},\pm g_{2},\ldots\pm g_{1}g_{2},\pm g_{1}g_{3},\ldots,\pm g_{1}g_{2}\ldots g_{N-1} \right]$.

For the calculation of generalized quaternion charges within our studied bi-anisotropic metamaterials, we first rotate the electromagnetic basis to align the polarizations parallel/perpendicular to the *C*_2_*T*-invariant plane, unitary transformation *U* is taken according to,

$$UHU^{-1}\cdot U\varphi^{'}=\omega U\varphi^{'}$$

with *U* in the form of,

$$U=\left[ \begin{matrix} \frac{1}{\sqrt{2}} & \frac{1}{\sqrt{2}} & 0 & 0 & 0 & 0 & 0 & 0 & 0 & 0 \\ -\frac{i}{\sqrt{2}} & \frac{i}{\sqrt{2}} & 0 & 0 & 0 & 0 & 0 & 0 & 0 & 0 \\ 0 & 0 & 1 & 0 & 0 & 0 & 0 & 0 & 0 & 0 \\ 0 & 0 & 0 & 0 & 0 & 0 & \frac{1}{\sqrt{2}} & \frac{1}{\sqrt{2}} & 0 & 0 \\ 0 & 0 & 0 & 0 & 0 & 0 & -\frac{i}{\sqrt{2}} & \frac{i}{\sqrt{2}} & 0 & 0 \\ 0 & 0 & 0 & -\frac{i}{\sqrt{2}} & \frac{i}{\sqrt{2}} & 0 & 0 & 0 & 0 & 0 \\ 0 & 0 & 0 & \frac{1}{\sqrt{2}} & -\frac{1}{\sqrt{2}} & 0 & 0 & 0 & 0 & 0 \\ 0 & 0 & 0 & 0 & 0 & -i & 0 & 0 & 0 & 0 \\ 0 & 0 & 0 & 0 & 0 & 0 & 0 & 0 & -\frac{i}{\sqrt{2}} & -\frac{i}{\sqrt{2}} \\ 0 & 0 & 0 & 0 & 0 & 0 & 0 & 0 & \frac{1}{\sqrt{2}} & -\frac{1}{\sqrt{2}} \end{matrix} \right]$$

where the electric (*E* and *P*) and magnetic (*H* and *M*) basis components are also rearranged into separated halves. *H* and $\varphi^{'}$ are of the form derived in supplementary information 1.

The Hamiltonian can be block diagonalized after being squared (without changing the topology),

$$H^{2}U\varphi^{'}=\omega^{2}U\varphi^{'}$$

and a 5×5 Hamiltonian is derived to be,

$H_{5\times5}=\left[ \begin{matrix} \frac{4A^{2}k_{z}^{2}-2l^{2}-\left( k_{x}^{2}-2k_{x}k_{y}+k_{y}^{2}+2k_{z}^{2} \right)L}{4A^{2}\varepsilon_{b}-2\varepsilon_{b}L} & \frac{i\left( k_{x}+k_{y} \right)\left[ 2iAl+\left( k_{x}-k_{y} \right)L \right]}{4A^{2}\varepsilon_{b}-2\varepsilon_{b}L} & -\frac{\left( k_{x}+k_{y} \right)k_{z}}{\sqrt{2}\varepsilon_{b}} & \frac{\omega_{0}\sqrt{L}\left[ l-iA\left( k_{x}-k_{y} \right) \right]}{\sqrt{\varepsilon_{b}}\left( 2A^{2}-L \right)} & 0 \\ -\frac{i\left( k_{x}+k_{y} \right)\left[ 2iAl+\left( k_{x}-k_{y} \right)L \right]}{4A^{2}\varepsilon_{b}-2\varepsilon_{b}L} & \frac{4A^{2}\left( l^{2}+k_{z}^{2}L \right)-L\left[ 2l^{2}+\left( k_{x}^{2}+2k_{x}k_{y}+k_{y}^{2}+2k_{z}^{2} \right)L \right]}{2\varepsilon_{b}\left( 2A^{2}-L \right)L} & \frac{i\left( k_{x}-k_{y} \right)k_{z}}{\sqrt{2}\varepsilon_{b}} & \frac{\omega_{0}\sqrt{L}A\left( k_{x}+k_{y} \right)}{\sqrt{\varepsilon_{b}}\left( 2A^{2}-L \right)} & -\frac{\omega_{0}l}{\sqrt{\varepsilon_{b}L}} \\ -\frac{\left( k_{x}+k_{y} \right)k_{z}}{\sqrt{2}\varepsilon_{b}} & -\frac{i\left( k_{x}-k_{y} \right)k_{z}}{\sqrt{2}\varepsilon_{b}} & \frac{k_{x}^{2}+k_{y}^{2}}{\varepsilon_{b}} & 0 & 0 \\ \frac{\omega_{0}\sqrt{L}\left[ l+iA\left( k_{x}-k_{y} \right) \right]}{\sqrt{\varepsilon_{b}}\left( 2A^{2}-L \right)} & \frac{\omega_{0}\sqrt{L}A\left( k_{x}+k_{y} \right)}{\sqrt{\varepsilon_{b}}\left( 2A^{2}-L \right)} & 0 & -\frac{\omega_{0}^{2}L}{2A^{2}-L} & 0 \\ 0 & -\frac{\omega_{0}l}{\sqrt{\varepsilon_{b}L}} & 0 & 0 & \omega_{0}^{2} \end{matrix} \right]$

It is apparent that the Hamiltonian is real at the *C*_2_*T*-invariant plane (*k_x_* = *k_y_*). We can thus calculate the generalized quaternion charges *n*_Г_ based on the equations above.

We then numerically calculated the generalized quaternion charges at the *C*_2_*T*-invariant plane. A set of gamma matrices in 5-dimensional space is adopted specifically as:

$\Gamma_{0}=\left[ \begin{matrix} I & 0 \\ 0 & -I \end{matrix} \right]$,$\Gamma_{1}=\left[ \begin{matrix} 0 & i\sigma_{1} \\ -i\sigma_{1} & 0 \end{matrix} \right]$,$\Gamma_{2}=\left[ \begin{matrix} 0 & i\sigma_{2} \\ -i\sigma_{2} & 0 \end{matrix} \right]$,$\Gamma_{3}=\left[ \begin{matrix} 0 & i\sigma_{3} \\ -i\sigma_{3} & 0 \end{matrix} \right]$,$\Gamma_{4}=\left[ \begin{matrix} 0 & I \\ I & 0 \end{matrix} \right]$

where *σ_i_* are Pauli matrices and *I* is the identity matrix.

Three loops are shown in Fig. S3 as green circles. In Fig. S3a, a loop is taken to encircle the chain point, by using the equations above, a charge of *n*_Г1_ = -1 is found. For the second loop in Fig. S3b, a charge of *n*_Г2_ = -g_2_ is found. From the definition of *g_j_* = *e_j_*_-1_·*e_j_*, we can infer that the -*g*_2_ charge corresponds to a band crossing between the 2^nd^ and 3^rd^ bands, which is consistent with the band structures shown in main text. We note that there is a zero-frequency band solution from the 5-dimensional Hamiltonian and is indexed as 0^th^ band and the index of *n*_Г_ = *g_j_* is also subtracted by one for convenience. We further check the loop shown in Fig. S3c, which can be seen as the joined loop of the loops in Fig.S3a and b, the charge is calculated to be *n*_Г3_ = *g*_2_, as also can be inferred from *g*_2_ = (-1)·(-*g*_2_) with group elements multiplication.


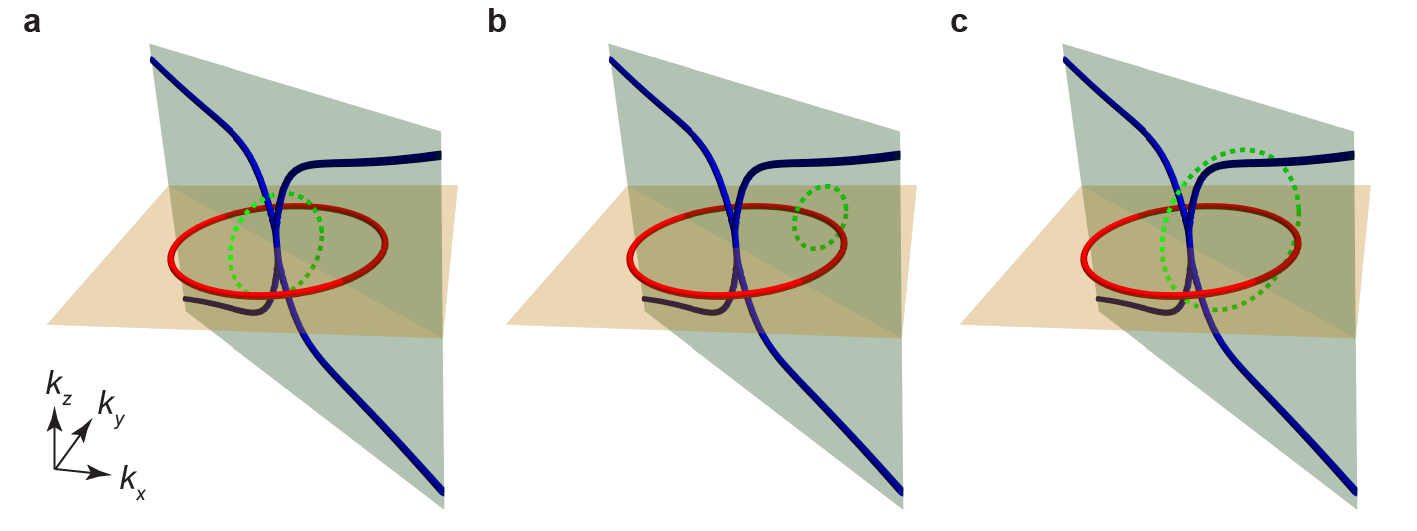


**Fig. S3 Non-Abelian generalized quaternion charges in bi-anisotropic metamaterials. a**. Green loop encircling the in-plane chain point gives a charge of -1. **b**. The loop containing a node from nodal ring gives a charge of -g_2_ indicating the band crossing between the 2^nd^ and 3^rd^ bands. **c**. A joined loop of **a** and **b**, where a charge of *g*_2_ is calculated, as can also be predicted from *g*_2_ = (-1)·(-*g*_2_).

**6. Rotation of eigen polarization states on *C*_2_*T*-invariant plane**

The generalized quaternion charges are frame rotation charges, which are related to the rotation of eigen polarization states. We show here the manifestation of polarization state rotation for all three scenarios of effective medium model discussed in Fig. 2 of main text. As shown in Fig. S4a and d, when no plasma resonance is introduced to gap the Г point, the green circle encircling the chain point corresponds to a generalized quaternion charge of n_Γ_ = -1, and correspondingly, the normalized projection of eigen polarization states ([*E*_+45˚_, *E_z_*] for the 2^nd^ band) on the *C*_2_*T-*invariant plane rotate by an angle of 2π. For the orange loop, a charge of g_2_ can be calculated which corresponds to the degeneracy between the 2^nd^ and 3^rd^ bands, as a consequence, the eigen states along the orange loop in Fig. S4d rotate by an angle of π.

If plasma resonances along x and y directions are introduced, a new nodal ring (blue) shows up in Fig. S4b as a result of conversation of the non-abelian charge of -1, which forbids the chain point to gap along *k*_-45˚_ direction. The polarization states in Fig. S4e also show that the winding phase along the green circle is still 2π. The orange loops again indicate winding phases of π. In Figs. S4c and f, the plasma resonance is introduced along z direction, the chain point is allowed to gap along *k_z_* direction, since the green loop in Fig. S4c is still encircling the two branches of the nodal chain. And in Fig. S4f, the winding phase along the green loop can again be found to be 2π, the orange loops are found with π winding phases.


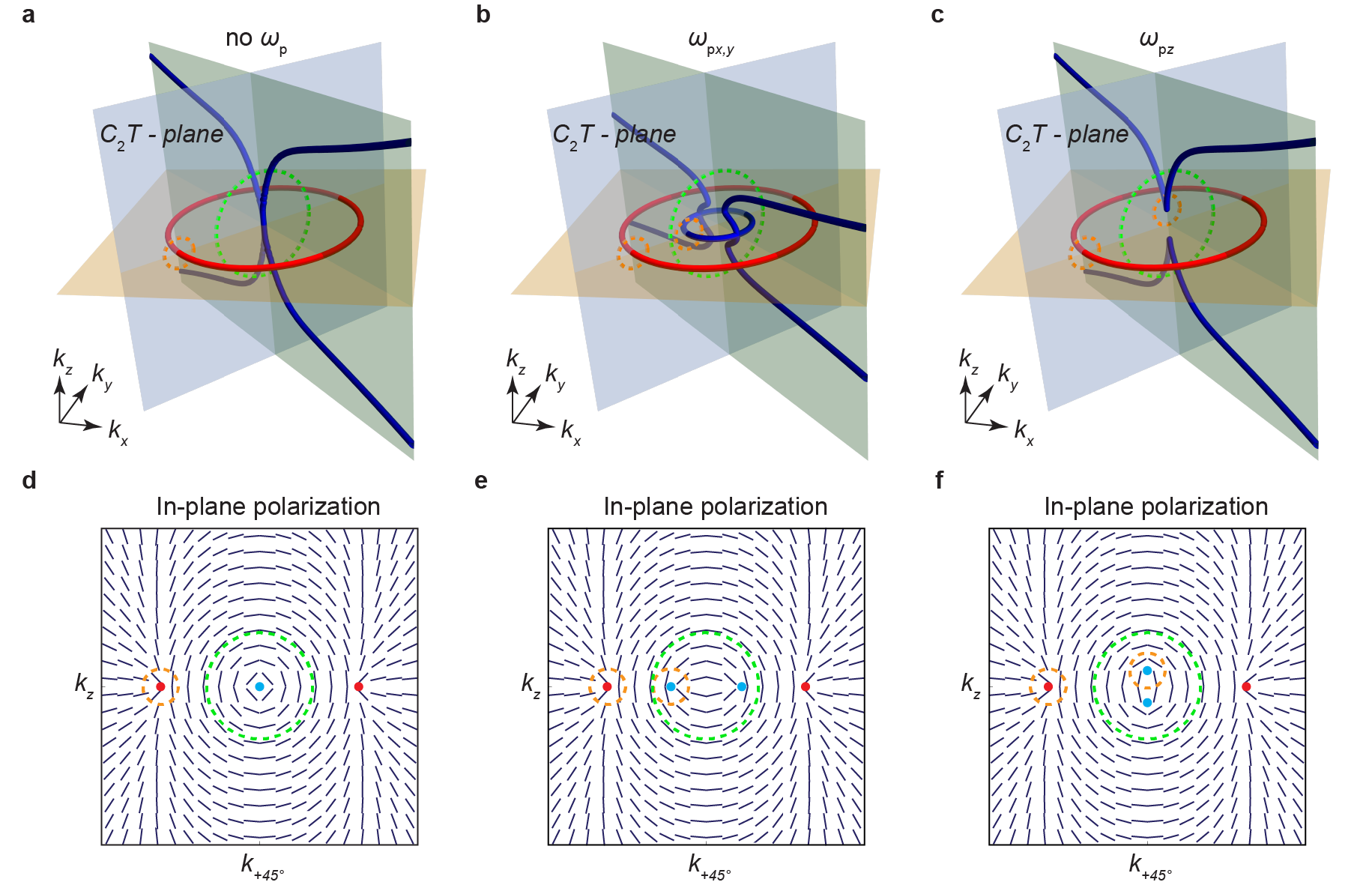


**Fig. S4 Manifestation of non-Abelian charges** **as rotation of eigen polarization states on *C*_2_*T*-invariant plane. a-c**. Nodal link from bi-anisotropic medium model with introducing no/x and y directions/z direction plasma resonance, respectively. Green and orange circles are the π_1_ homotopy loops, the green loops correspond to generalized quaternion charge of -1, and the orange ones correspond to charges of *g_j_*, as indications of degeneracies between the j^th^ and (j+1)^th^ bands. **d-f**. The normalized projection of eigen polarization states on *C*_2_*T*-invariant plane. Green circles indicate winding phases of 2π, orange circles indicate the winding phases of π. Red dots are the intersections of *C*_2_*T*-invariant plane with nodal ring (red ring in **a**-**c**). Blue dots are the intersections of *C*_2_*T*-invariant plane with blue nodal curves in **a**-**c**.

**7. Surface modes at side surfaces**

By placing the source antenna position close to the interface, surface modes can be excited more efficiently. We thus experimentally measured the surface modes for the side surfaces as supplement to the bulk properties discussed in main text. The experimental configuration is shown in Fig. S5a, where the top side (parallel with resonator gap) and bottom side are indicated. In Fig. S5b, calculated results of projected bands are shown, where three branches of surface modes are marked with different colors. The red and orange branches are found to be localized at the top surface, and the blue branch is localized at the bottom surface. It can be seen that the orange and blue branches connect to the projection of band degeneracy of in-plane nodal chain (blue dot), which indicates that the surface modes originate from the line nodes of in-plane nodal chain. Meanwhile, the red branch can be found to merge into the projected bulk bands of nodal ring frequencies (red dashed line), which indicates that the surface mode is induced by the nodal ring.

We then show the experimentally measured surface modes at the top side and bottom side. The results are shown in Figs. S5c and d, where the predicted surface modes are also shown. It can be seen in Fig.S5c and d that the two surface modes connecting to blue dot are slightly shifted away from predicted positions (white dashed curves). The frequency shift can be attributed to the difference of geometrical parameters between the fabricated sample and simulations, where the sensitivity of surface modes with interface condition leads to the mismatch.


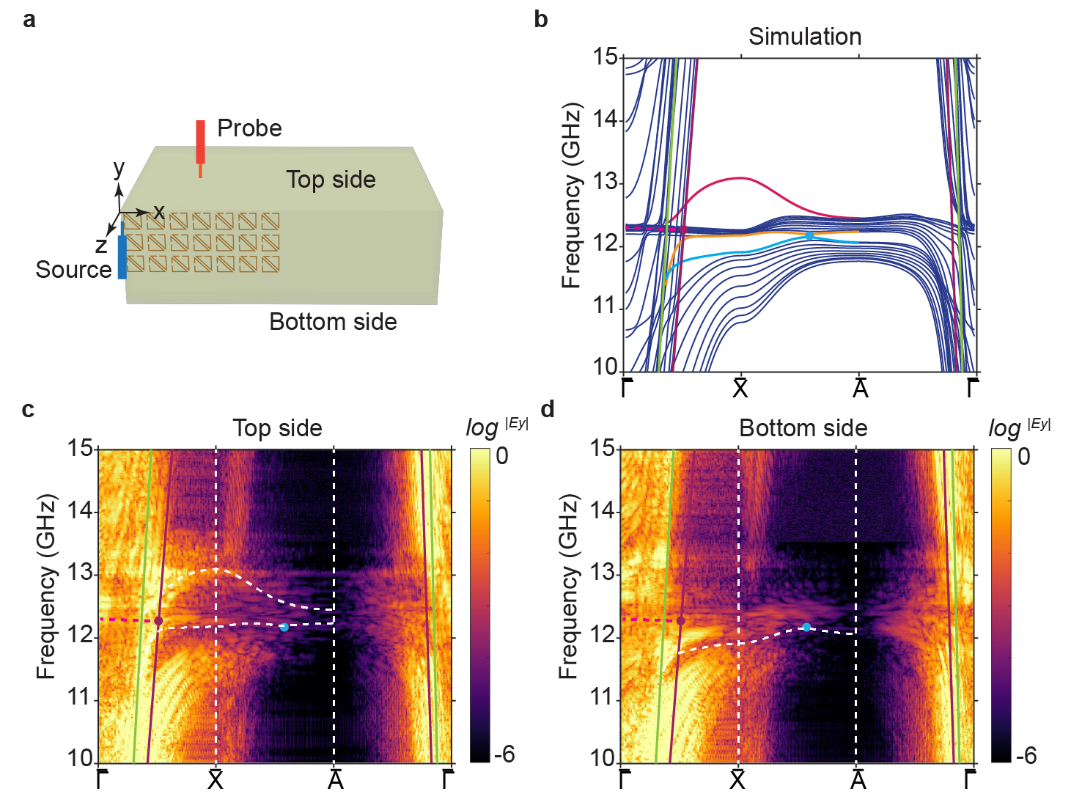


**Fig. S5 Surface modes at opposite interfaces of side-arrayed sample.** **a**. Experimental setup for surface modes measurement of side surfaces (*x* - *z* plane). **b**. Calculated results for projected bands for side surface configuration. Three surface modes can be found at the two opposite interfaces of sample, where the red and orange ones are located at the top side and the blue one at the bottom side. Green and red solid lines are light cones for air and substrate, respectively. **c**. Experimentally measured surface modes at top side, where the source antenna is put at position closed to the interface. Two surface modes are indicated with white dashed curves. **d**. Experimentally measured surface modes at bottom side with similar source antenna arrangement. Surface mode is indicated with white dashed curve again.

**8. Role of bi-anisotropic couplings**

The bi-anistropic couplings break the inversion symmetry of the photonic system, which establish the minimal model with symmetry conditions that support in-plane nodal chain and nodal link to show up. Here, we can further check the role played by bi-anisotropic terms, by comparing different effective medium models as following cases.

Case 1: Simple electric resonance,

$\varepsilon=\left[ \begin{matrix} \varepsilon_{x} & 0 & 0 \\ 0 & \varepsilon_{y} & 0 \\ 0 & 0 & \varepsilon_{b} \end{matrix} \right]$, $\mu=\left[ \begin{matrix} 1 & 0 & 0 \\ 0 & 1 & 0 \\ 0 & 0 & 1 \end{matrix} \right]$,

with $\varepsilon_{x}=\varepsilon_{y}=\varepsilon_{b}+\frac{1}{\omega_{0}^{2}-\omega^{2}}\frac{l^{2}}{L}$, the band dispersions and nodal structures in momentum space is shown in Fig. S6a, where a nodal ring in linked with two nodal lines.

Case 2: Electric and magnetic resonance

$\varepsilon=\left[ \begin{matrix} \varepsilon& 0 & 0 \\ 0 & \varepsilon& 0 \\ 0 & 0 & \varepsilon_{b} \end{matrix} \right]$, $\mu=\left[ \begin{matrix} 1 & 0 & 0 \\ 0 & 1 & 0 \\ 0 & 0 & \mu\end{matrix} \right]$,

with $\varepsilon_{x}=\varepsilon_{y}=\varepsilon_{b}+\frac{1}{\omega_{0}^{2}-\omega^{2}}\frac{l^{2}}{L}$, $\mu_{z}=1+\frac{2}{\omega_{0}^{2}-\omega^{2}}\frac{\omega^{2}A^{2}}{L}$, the band dispersion and nodal structures in momentum space are shown in Fig. S6b, where a nodal ring is linked with two nodal lines, in addition, there is a nodal surface induced by the duality symmetry of EM wave and emerges when $\frac{\varepsilon}{\varepsilon_{b}}=\frac{1}{\mu}$. There is also a pair of triple degeneracy points indicated with red dots in Fig. S6b (first panel).

Case 3: Electric and magnetic resonance with bi-anisotropic couplings

$\varepsilon=\left[ \begin{matrix} \varepsilon& 0 & 0 \\ 0 & \varepsilon& 0 \\ 0 & 0 & \varepsilon_{b} \end{matrix} \right]$, $\mu=\left[ \begin{matrix} 1 & 0 & 0 \\ 0 & 1 & 0 \\ 0 & 0 & \mu\end{matrix} \right]$, $\varsigma=\left[ \begin{matrix} 0 & 0 & 0 \\ 0 & 0 & 0 \\ -i\chi& -i\chi& 0 \end{matrix} \right]$, $\xi=\left[ \begin{matrix} 0 & 0 & i\chi\\ 0 & 0 & i\chi\\ 0 & 0 & 0 \end{matrix} \right]$

with $\varepsilon=\varepsilon_{b}+\frac{1}{\omega_{0}^{2}-\omega^{2}}\frac{l^{2}}{L}$, $\mu=1+\frac{2}{\omega_{0}^{2}-\omega^{2}}\frac{\omega^{2}A^{2}}{L}$, $\chi=\frac{\omega}{\omega_{0}^{2}-\omega^{2}}\frac{Al}{L}$. In this case, there is a nodal ring enclosing the in-plane nodal chain, which is a clean and simple model of in-plane nodal chain and nodal link shown in Fig. S6c.

It is worth to note that the proposed in-plane nodal chain (even the nodal link) widely exists in photonic systems as we pointed out in main text. For the cases discussed here, the in-plane nodal chain and nodal link can also be found in case 1 and 2. The nodal lines in both cases can be examined to be quadratic, by breaking the symmetry of *ε_x_* = *ε_y_* symmetry, the quadratic nodal line will split into in-plane nodal chain which will be discussed with more details in future work.


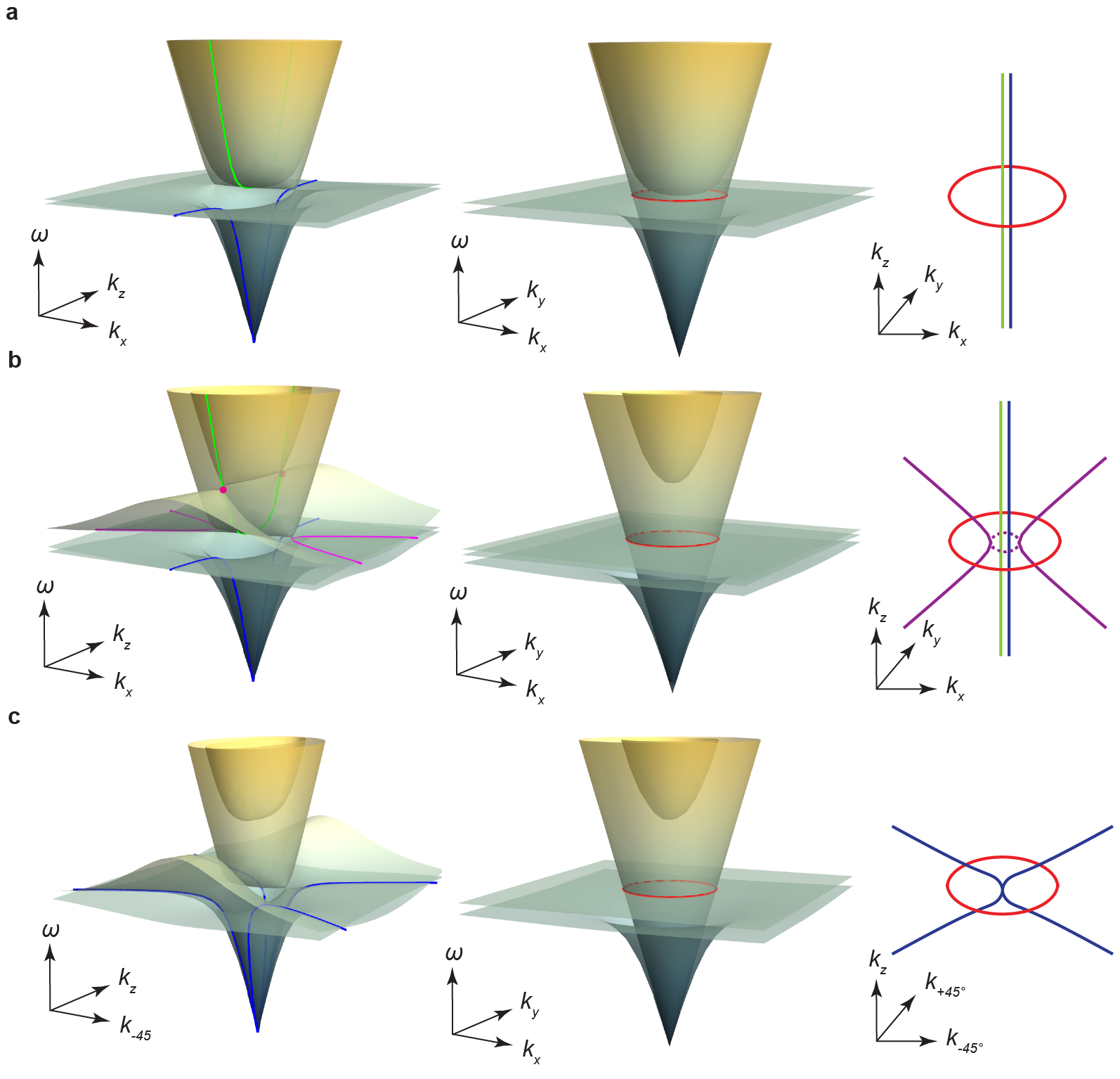


**Fig. S6 Role of bi-anisotropic in in-plane nodal chain and nodal link.** **a**. Bulk bands and nodal structures in simple electric resonance material with effective parameters of [*ε_x_*, *ε_y_*, *ε*_b_], there are two quadratic nodal lines (green and blue colors) encircled by nodal ring (red color) to form nodal link. **b**. Bulk bands and nodal structures with additional magnetic resonance of *μ_z_*, more topological structures of nodal surface (purple color) that originates from EM duality symmetry and triple degeneracy point (marked with red dots) are shown. **c**. Effective medium with bi-anisotropic couplings gives clean in-plane nodal chain as well as nodal link in momentum space.

**9. Electromagnetic duality symmetry of bi-anisotropic metamaterials**

Due to the EM duality symmetry, another configuration of bi-anisotropic couplings can give the same topological structures of in-plane nodal chain and nodal link. We consider the metamaterials structure shown in Fig. S7a, the corresponding effective constitutional parameters are:

$\varepsilon=\left[ \begin{matrix} \varepsilon_{b} & 0 & 0 \\ 0 & \varepsilon_{b} & 0 \\ 0 & 0 & \varepsilon\end{matrix} \right]$, $\mu=\left[ \begin{matrix} \mu& 0 & 0 \\ 0 & \mu& 0 \\ 0 & 0 & 1 \end{matrix} \right]$, $\varsigma=\left[ \begin{matrix} 0 & 0 & -i\chi\\ 0 & 0 & -i\chi\\ 0 & 0 & 0 \end{matrix} \right]$, $\xi=\left[ \begin{matrix} 0 & 0 & 0 \\ 0 & 0 & 0 \\ i\chi& i\chi& 0 \end{matrix} \right]$

with,

$\varepsilon=\varepsilon_{b}+\frac{2}{\omega_{0}^{2}-\omega^{2}}\frac{l^{2}}{L}$,$\mu=1+\frac{1}{\omega_{0}^{2}-\omega^{2}}\frac{\omega^{2}A^{2}}{L}$,$\chi=\frac{\omega}{\omega_{0}^{2}-\omega^{2}}\frac{Al}{L}$.

The effective model gives nodal structures shown in Fig. S7b, where again the nodal ring as well as in-plane nodal chain are found as expected.

The analytical descriptions of nodal curves can also be found. The two crossing bands of nodal ring are,

$\omega_{Ring}^{1}=\frac{\sqrt{k_{x}^{2}+k_{y}^{2}}}{\sqrt{\varepsilon_{b}}}$,$\omega_{Ring}^{2}=\frac{\sqrt{L}\omega_{0}}{\sqrt{L-A^{2}}}$,

which gives the solution of nodal ring as,

$k_{x}^{2}+k_{y}^{2}={\varepsilon_{b}\omega_{0}^{2}L}/\left( L-A^{2} \right)$.

The nodal ring is shown in Fig. S7b as a red circle.

For the in-plane nodal chain, we have,

$\omega_{Nc}^{1}=\frac{\sqrt{2\varepsilon_{b}k_{x}^{2}+\varepsilon k_{z}^{2}}}{\sqrt{\varepsilon_{b}\left( \varepsilon\mu-2r^{2} \right)}}$, $\omega_{Nc}^{2}=\frac{\sqrt{2\mu k_{x}^{2}+k_{z}^{2}}}{\sqrt{\varepsilon_{b}\mu}}$

The nodal chain can be found to be,

$$k_{z}^{2}=\frac{k_{x}\left( A^{2}k_{x}^{2}+l^{2} \right)}{2A^{2}l^{2}\left( l^{2}+k_{x}^{2}L \right)}\left( -2k_{x}^{2}l^{2}L+A^{2}k_{x}\left( 2l^{2}+\varepsilon_{b}L\omega_{0}^{2} \right)\pm\sqrt{4k_{x}^{2}l^{4}L^{2}+A^{4}k_{x}^{2}\left( 2l^{2}+\varepsilon_{b}L\omega_{0}^{2} \right)^{2}+4A^{2}l^{2}L\left( 2\varepsilon_{b}l^{2}\omega_{0}^{2}+k_{x}^{2}\left( \varepsilon_{b}L\omega_{0}^{2}-2l^{2} \right) \right)} \right)$$

The in-plane nodal chain is plotted in Fig. S7b in blue color. The periodically arrayed units give first Brillouin zone in Fig. S7c. The band structures along indicated trajectories are calculated as shown in Fig. S7d. The ring degeneracies can be verified along Γ-X and Γ-Mʹ directions, and the band degeneracy for in-plane nodal chain can be found along Mʹ-A line.


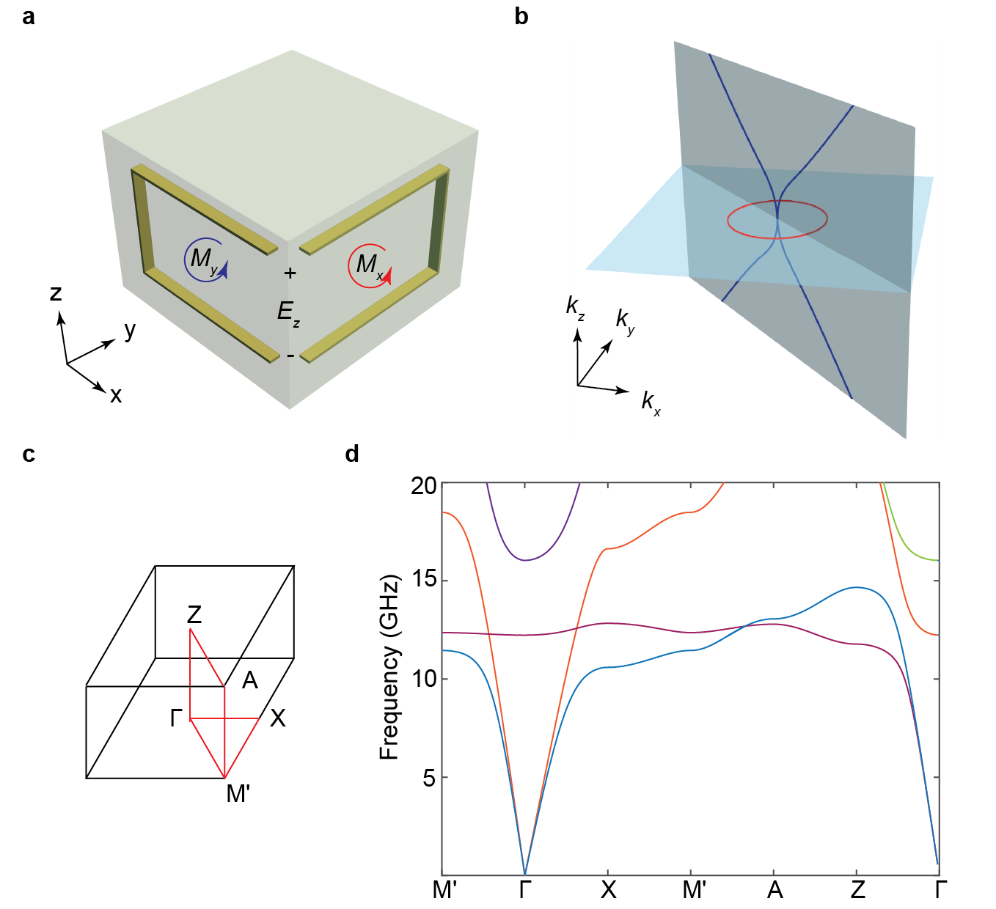


**Fig. S7 Duality configuration of bi-anisotropic metamaterials.** **a**. Bi-anisotropic metamaterials design with *E_z_* coupled to *M_x_* and *M_y_*. **b**. Nodal structures calculated with effective medium model, where an in-plane nodal chain and a nodal ring are again found to form into nodal link. **c**. Brillouin zone and indicated high symmetry lines in momentum space. **d**. Calculated band structure for real metamaterials, where the nodal ring and in-plane nodal chain degeneracies are verified as band crossings along Γ-X(Mʹ) and Mʹ-A, respectively.

**10. In-plane nodal chain transformation with mirror symmetry breaking**

The in-plane nodal chain is protected by the mirror symmetry *σ*_-45˚_, a tuning of two resonators’ size as *l*_1_, *A*_1_ and *l*_2_, *A*_2_ will break the nodal lines into Weyl points. In this case, the effective parameters are,

$\varepsilon=\left[ \begin{matrix} \varepsilon_{1} & 0 & 0 \\ 0 & \varepsilon_{2} & 0 \\ 0 & 0 & \varepsilon_{b} \end{matrix} \right]$, $\mu=\left[ \begin{matrix} 1 & 0 & 0 \\ 0 & 1 & 0 \\ 0 & 0 & \mu\end{matrix} \right]$, $\varsigma=\left[ \begin{matrix} 0 & 0 & 0 \\ 0 & 0 & 0 \\ -i\chi_{1} & -i\chi_{2} & 0 \end{matrix} \right]$, $\xi=\left[ \begin{matrix} 0 & 0 & i\chi_{1} \\ 0 & 0 & i\chi_{2} \\ 0 & 0 & 0 \end{matrix} \right]$

with,

$\varepsilon_{1,2}=\varepsilon_{b}+\frac{1}{\omega_{0}^{2}-\omega^{2}}\frac{l_{1,2}^{2}}{L}$, $\mu=1+\frac{\omega^{2}}{\omega_{0}^{2}-\omega^{2}}\frac{A_{1}^{2}+A_{2}^{2}}{L}$, $\chi_{1,2}=\frac{\omega}{\omega_{0}^{2}-\omega^{2}}\frac{A_{1,2}l_{1,2}}{L}$

We consider the case of *l*_1_ = 0.5, *l*_2_ = 1.5, *A*_1_ = *A*_2_ = 0.5, and calculate the Berry curvature of four bands as shown in Fig. S8. We can see in Fig. S8a and b that the in-plane nodal chain transforms into four Weyl points when the mirror symmetry is broken. Each pair of Weyl points are associated with time reversal symmetry and carry the same topological charges. The two pairs of Weyl points are of opposite charges and lead to the total charge of zero.


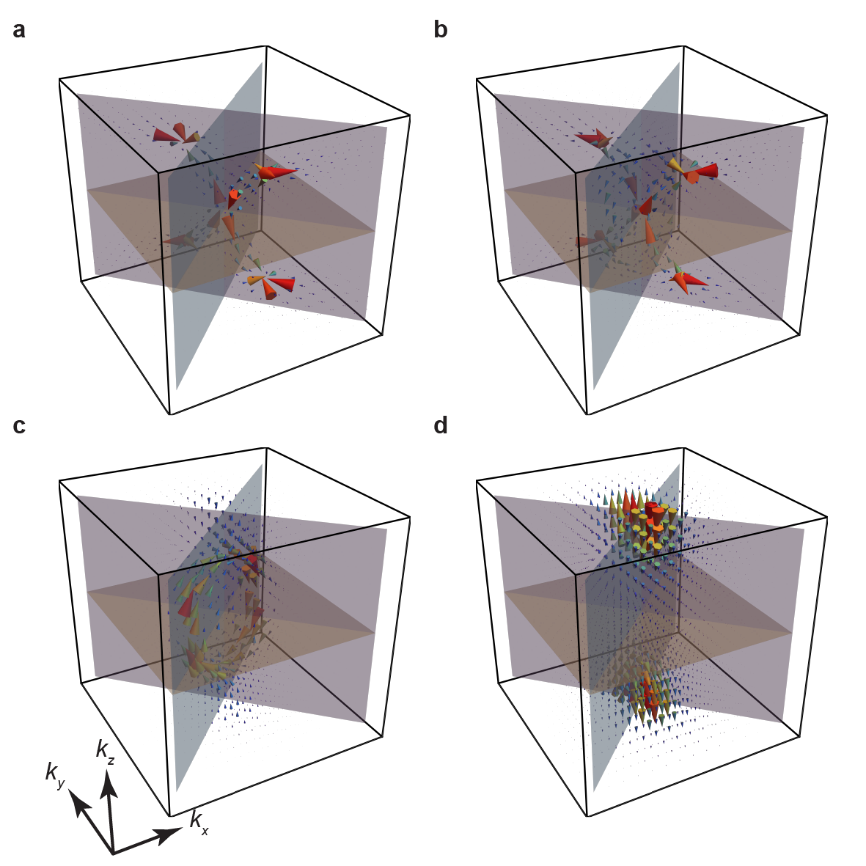


**Fig. S8 Berry curvature for different bands with broken *σ*_-45°_ mirror symmetry.** **a**. For the 1^st^ band, four sources and sinks of Berry curvature can be found closed to the *σ*_-45°_ plane, which indicate that the in-plane nodal chain (degeneracies between the 1^st^ and 2^nd^ bands) transforms into four Weyl points. **b**. Two sources and two sinks show up for 2^nd^ band as well. Additionally, toroidal shape Berry curvature can also be seen. **c**. For the 3^rd^ band, a toroidal shape distribution of Berry curvature overlaps with the u-shape one. **d**. U-shape Berry curvature is found for 4^th^ band. The total Berry curvature for the 4 bands neutralizes.
